# Diagnosing callous-unemotional personality traits by heart rate orienting responses to images inducing threat and distress

**DOI:** 10.1038/s41598-023-49307-7

**Published:** 2023-12-12

**Authors:** Günter Schulter, Beatrice Milek, Helmut Karl Lackner, Bernhard Weber, Andreas Fink, Christian Rominger, Corinna Perchtold-Stefan, Ilona Papousek

**Affiliations:** 1 Biological Psychology Unit, Department of Psychology, University of Graz, Graz, Austria; 2 Otto Loewi Research Center, Division of Physiology, Medical University of Graz, Graz, Austria

**Keywords:** Neuroscience, Physiology, Psychology

## Abstract

The present study aimed at developing a rather easily applicable method of testing physiological reactions to images of threats and misery. To this end, rapid-changing, transient heart rate orienting responses were used for gaining physiologically based, objective responses to the images. Additionally, subjective ratings were obtained. A significant insensitivity to other's welfare and well-being was already demonstrated as a core feature of callous-unemotional personalities. Thus, physiologically based methods may supplement and possibly improve existing assessments and, in particular, may contribute to a multimodal assessment of psychopathic traits. Out of a non-forensic community sample of 122 men, we selected two extreme groups of 30 participants with the lowest and highest callous-unemotional traits respectively, ascertained by questionnaires. As expected, participants with higher scores of callous-unemotional traits showed smaller responses to distress cues in both heart rate responses and subjective ratings. Moreover, within the group with high callous-unemotional traits heart rate responses to threatening as well as distress cues did not significantly differ from responses to neutral pictures. The study provides further evidence for the idea that a lack of responsiveness to distress cues may be seen as a central feature of callous-unemotional personalities.

## Introduction

The construct of psychopathy is seen as a constellation of personality characteristics comprising several features. At the core of the heterogeneous concept is a disturbance in the affective domain, characterized by shallow or deficient affect. Associated traits are interpersonal and behavioral features such as deceitfulness and social deviance, most often resulting in serious criminality and certain lifestyle dispositions such as grandiosity and superficial charm^1^. While there is consensus on the pertinence of psychopathy in clinical and forensic settings, the conceptual breadth and boundaries of the condition are still debated^2^. As regards the nature of the construct, for instance, there is a variety of evidence that individual differences in psychopathic traits are continuously distributed in the general population. Psychopathic personality, therefore, is seen as dimensional in nature, while the syndrome of psychopathy is a configuration of extreme levels of continuously distributed personality traits^3,4^. The dimensionality of psychopathic traits was demonstrated in a variety of ethnic and community samples, comparing prisoners, community adults, university students, adolescents, and also men and women in a general population sample, to investigate differences and similarities across genders^5–7^. Consequently, diagnostic procedures, especially self-report questionnaires were developed to be used in community samples, in which specific items relevant to criminal populations were excluded. Due to several core features of a psychopathic personality (e.g., lying, deception/manipulation), there is some concern that self-report measures may not be valid indicators of psychopathic traits. However, while a particularly high amount of faking might be expected, especially in forensic settings, some studies indicate a sufficient validity of self-report measures^8,9^ for the assessment of psychopathic traits, nevertheless.

In line with a dimensional conceptualization of psychopathy, the present study used a community sample to develop a new diagnostic procedure that is based on physiological responses to specific stimuli. The purpose of this development was to contribute to a multimodal assessment of psychopathic personality traits in individuals who do not yet stand out, e.g., for previous criminal convictions. As our study is the first test of this psychophysiological approach, we confined it to only one specific facet of psychopathy, i.e., the callous-unemotional (CU) trait. Of course, this is a severe limitation in terms of the method's possible diagnostic value, because in most cases psychopathic behavior is determined by various facets. A callous intention to commit a criminal act, for instance, is more likely to be put into practice, if one is also able to manipulate a victim and does not fear an impending punishment. Consequently, specific constellations of psychopathic traits may be conducive concerning a specific criminal behavior as was recently demonstrated, for example, by Colins and coworkers^6^.

One of the most prominent characteristics of psychopathic personalities is deficiencies in the affective domain. The callous-unemotional trait may be seen as the basis for several problematic behaviors such as uncaring and antisocial behavior, which are additionally accompanied by a lack of remorse or guilt. For instance, poor autonomic fear conditioning, a related and well-replicated correlate of psychopathic personality, is already indicative of an increased predisposition to adult criminal activity when observed in 3 years old children^10^. A deficient negative emotional arousability seems to be the essential neurobiological constraint for the development of fundamental moral emotions and may thereby lay the foundation for a callous-unemotional personality. Recent observations of an inverse relationship between self-reported callous-unemotional traits and fear conditioning lent support to this assumption^11^.

Without a doubt, there is a marked tendency of psychopathic personalities to frequently and severely violate social norms. Consequently, psychopathy is seen as a mental disorder, and psychopathic personality traits as highly maladaptive. However, there is growing evidence that there may also be benefits of certain characteristics of psychopathy. For instance, higher levels of ‘unemotionality’ may be associated with reduced experience of fear and anxiety and, more generally, with greater resilience to stress. Therefore, the consequences of maltreatment in childhood for the development of callous-unemotional behaviors are ambiguous, and it is clear that genes and environment interact^12,13^. Furthermore, complex interactions between parenting style, psychophysiological responsivity, and callous-unemotional traits were reported. For example, higher levels of authoritarian parenting were associated with lower resting heart rate in their children, as well as with higher levels of callous-unemotional traits^14^. Furthermore, the association between heart rate reactivity in a stressor task and callous-unemotional traits depended on a history of paternal or maternal maltreatment in girls^15^. Moreover, there is evidence that children with high levels of callous-unemotional traits show little eye contact with their mothers, which is a non-adaptive strategy in a social environment. This habit was associated with maternal reports of negative feelings towards the children but also with psychopathic fearlessness in their fathers. This may corroborate the interactive effects of genes and complex family conditions on the development of psychopathic traits in children^16^.

All the more, diagnosing psychopathic traits as reliably and accurately as possible continues to be an important task. At present, the most widely used instrument to assess psychopathy, especially in forensic settings, is the Psychopathy Checklist-Revised (PCL-R)^17,18^. However, this diagnostic tool is subject to criticism. In particular, in the PCL-R diagnostics crucial deficiencies in the affective domain, manifesting in a lack of empathy and a callous-unemotional personality are only indirectly inferred by the forensic experts in PCL-R diagnostics^19,20^. However, forensic health professionals rated items such as ‘callous/lack of empathy’ as the most prototypical features of psychopathy^21,22^. Therefore, completion of the PCL-R diagnostic seems required.

Furthermore, a multimodal approach would add benefit to the diagnosis of psychopathic personalities. The use of psychophysiological parameters was repeatedly advocated as a possible and very valuable supplement to self- or other-reported questionnaires. It establishes a greater focus on measuring the neurobiological underpinnings of psychopathic personality, thereby providing a better understanding of etiological aspects^23,24^. Consequently, a well-manageable neurophysiologically based test procedure may be a useful complement to any assessment of psychopathic traits.

In previous research, a great variety of physiological parameters were related to various facets of psychopathic personalities. Startle, skin conductance, and cardiac responses but also certain EEG parameters (late positive potentials, theta coherence) were linked to affective deficits in participants with psychopathic traits. Associated experimental settings vary in effort and feasibility, with EEG parameters requiring the most expensive and laboratory-intensive facilities. On the other hand, due to their fast habituation skin conductance measures are greatly limited in the repeatability of test trials. For these reasons, heart rate responses seem to be most qualified for the assessment of arousability and emotion processing also outside specialized laboratories.

Several studies investigated cardiac measures in individuals with callous-unemotional traits. Various heart rate parameters were used, e.g., heart rate at rest, heart rate in the aftermath of stimulation with various external stimuli (pictures, film clips, acoustic stimulation, etc.), and heart rate reactivity, i.e., the change from a resting state to specific stimulation. So far, studies using measures of callous-unemotional traits in combination with cardiac measures have provided inconsistent results. Some reported complex associations between trait variables, physiological measures, and participant variables such as age and sex. For instance, in a community sample of juveniles, an inverse relationship between resting heart rate and callous-unemotional traits was observed^25^. But in a large community sample of undergraduate students the same group of researchers reported little support for an association between heart rate at rest or in response to a stressor and callous-unemotional traits^26^. Callous-unemotional traits were also not related to resting heart rate or heart rate reactivity to violent and erotic movie scenes but were only negatively associated with startle potentiation in response to violent scenes^27^. In a similar study in samples of adolescents and young adults, low startle potentiation for violent stimuli was again related to callous-unemotional traits and meanness observed by the Triarchic Psychopathy Measure. However, a lower resting heart rate and cardiac reactivity to violent stimuli were only associated with increased boldness^28^.

Other studies on callous-unemotional traits reported interesting but complex results if additional traits were included. In a large community sample of children, for instance, callous-unemotional traits were associated with greater heart rate reactivity to fear stimuli only when autistic traits were low^29^. A recent study reported an interesting but also a complex interaction between resting heart rate, callous-unemotional traits, and the likelihood of offence, depending on the participants' age^30^. High levels of callous-unemotional traits in connection with low heart rates in early adolescents and high heart rates in late adolescents were associated with the highest likelihood of offence. Moreover, a recent relevant meta-analysis including various diagnostic instruments reported very heterogeneous effect sizes of heart rate and skin conductance measures. The authors concluded that the careful partitioning of variables may shed light on the complex moderation of effects by the experimental tasks used, parameters calculated, and analyses run. Most importantly, the authors suggested that the use of highly specific psychophysiological responses may be expedient to differentiate between (and within) types of antisocial behavior^31^. In a recent review of psychophysiological studies on conduct disorder, the author also underlined the heterogeneity of results and emphasized the importance of investigating the associations between specific psychopathic traits and relevant responses in specific physiological systems. In a similar vein, he recommended the use of well-reasoned psychophysiological variables for the potential identification of distinct diagnostic categories or subtypes of specific psychopathic phenomena and traits^32^.

Deficits in empathic fear recognition were also observed in a series of studies using other research paradigms demonstrating, for instance, reduced facial affect recognition in antisocial individuals^33,34^. Particularly relevant to the present study, any acute distress in others should trigger emotional empathic responses and corresponding physiological alterations as the basis of stimulus-reinforcement learning in moral development^35^. In other words, the sensitivity to acute distress in others may be seen as a prerequisite for the development of caring behaviors and empathic concern with suffering fellow human beings. Therefore, an appropriate picture-viewing test concept for the diagnosis of callous-unemotional traits must not only include threatening items but also distress cues. Empirical evidence supports this notion: Blair and colleagues^36^ concluded that the lack of responsiveness to cues indicating distress of others is one of psychopathic individuals’ main deficiencies. Remarkably, such a callous lack of responsiveness to distress cues was demonstrated already in preschoolers: a fear-specific deficit in emotion recognition was significantly associated with parental ratings of a child’s insensitivity to the feelings of others, i.e., was related to the child's callous-unemotional traits^37^.

The present study aimed at developing a simple and easily applicable method of testing cardiac responses to cues of threat and others’ misery. As noted above, there is a large heterogeneity in the cardiac measures and experimental designs in psychophysiological studies on callous-unemotional traits. Heart rate at rest, for instance, was recorded in periods ranging from single events to long time intervals (e.g., 60 s and more). Interestingly, long recording periods do not appear to necessarily yield more consistent results than, for example, stimulation with single loud noise probes in the startle reflex paradigm, which has proven valid in two different studies^27,28^. Whereas cardiac measures averaged across extended recording periods or in resting conditions are evidently too unspecific for gaining indicators of specific personality dispositions, rapid-changing, transient heart rate responses to well-selected stimuli provide well-validated indicators of specific personality traits. Measures of this kind have already been used successfully for gaining physiologically based, objective responses to images of misery and threat in several previous studies of our laboratory^38,39^. The comparison of two non-forensic community samples, where differences in the relevant personality characteristics are much smaller compared to the testing of forensic versus non-forensic groups additionally calls for a particularly specific and sensitive measure.

In addition to a callous lack of responsiveness to distress cues, several other studies on the psychopathic personality reported deficiencies in the recognition and subjective experience of fear. Accordingly, a more detailed concept of different aspects of fear-related emotions and fearlessness was proposed recently^40^. The authors presented evidence for deficits in the responsivity to threatening stimuli and situations, and the subjective experience of fear. Our study design also allowed us to test these important aspects of deficient fear processing.

In brief, we presented images with threatening, distressing, and neutral content to participants with low and high callous-unemotional scores. As dependent measures we observed the immediate, rapid heart rate orienting responses to these stimuli as well as the participants’ subjective evaluation of the stimuli in three categories (‘The picture makes me sad/furious/afraid’). Participants represented a sample of young adults from the general population. As outlined below, the essential hypotheses of the study relate to specific two-way and three-way interactions, i.e., level of callous-unemotional traits by stimulus type and callous-unemotional traits by stimulus type by rating category. Consequently, the realization of a full-dimensional approach in the data analysis was not possible, and we compared extreme groups with low vs. high callous-unemotional traits. For the evaluation of two- and three-way interactions with a correlational strategy, a much larger sample size would be required.

Taken together, we expected, firstly, lower responsivity to threat, i.e., a smaller cardiac response to threatening stimuli in participants with higher compared to those with lower callous-unemotional scores. Secondly, we expected lower fear ratings of threatening stimuli, i.e., lesser subjective experience of fear in participants with higher callous-unemotional scores.

Concerning callousness, we expected a smaller cardiac response to distress cues in participants with higher compared to those with lower callous-unemotional scores. Finally, we expected lower impact scores, i.e., lower negative ratings of distress cues (sad, furious) in the callous-unemotional group compared to participants with low scores.

In addition, different responses to the different stimulus types are essential for the validity of our approach. Therefore, we also expected higher cardiac orienting responses to threatening as well as distress cues compared to neutral stimuli. Furthermore, these differences should be more/less pronounced in participants with low/high callous-unemotional scores, respectively, confirming the construct validity of our physiological test paradigm (interaction callous-unemotional by stimulus type). We expected that in participants with high callous-unemotional traits, the physiological responses to threatening and distress cues should be of comparable size as those occurring to neutral stimuli, whereas in participants with low scores, threat and distress cues would evoke more pronounced orienting responses than neutral cues.

## Methods and materials

### Participants

122 men from a non-forensic community sample participated in the study. Participants were mainly students (61%) from different subject areas but also people from various non-academic occupational backgrounds. The age range was 18–45 years (M = 26.3; SD = 4.9). The study was confined to men, as research indicated gender differences in the degree as well as the manifestation of callous-unemotional (CU) traits. Such differences may result both from gender differences in socialization processes as well as from biological diversity.

Exclusion criteria were neurological and cardiovascular disorders according to self-report (e.g., brain damage, cardiac arrhythmias) and medication for heart conditions. Moreover, individuals with a resting heart rate lower than 40 bpm and a blood pressure higher than 140/90 mmHg were excluded. Participants were asked to refrain from smoking for at least half an hour before testing, from alcohol for 12 h, and from stimulating beverages (coffee, Coca-Cola, energy drinks, etc.) for 2 h before testing. Participants received no payment for their participation. However, each participant took part in a draw for three “discount tickets”, each worth €10. As we used a non-forensic community sample, we selected two extreme groups with the highest and lowest CU scores, respectively, comprising 30 participants each.

The study was conducted in accordance with the Declaration of Helsinki, and the University Institutional Review Board, i.e., the Ethics Committee of the University of Graz, Austria (reference number GZ. 39/30/63 ex 2017/18) approved all study procedures. Informed written consent was obtained from all participants. Moreover, participants were informed about all details of the experiment at the end of the study.

### Inventory of callous-unemotional traits (ICU)

The ICU^41^—German version^42^—was used in its self-report version to assess one of the most prominent components of psychopathy, i.e., the callous and unemotional personality trait. The ICU was originally designed to assess CU traits in children and adolescents, but several studies have demonstrated the same factor structure and validity of the ICU in adult samples^43–45^. In analyzing the ICU data we did not use a unit-weighted total score summing up all of the items but did apply a bifactor model^46^, calculating separate scores for the three subscales Callous (11 items), Unemotional (5 items) and Uncaring (8 items). Internal consistencies (Cronbach’s Alpha) in our sample were 0.72, 0.81, and 0.71, respectively.

### The personality inventory for DSM-5 (PID-5; adult)

This inventory is based on an alternative model for personality disorders in the Diagnostic and Statistical Manual of Mental Disorders (DSM-5, Section III)^47–49^. Participants filled in the complete questionnaire consisting of 220 items assessing a total of 25 personality trait facets and five broader trait domains.

### Defining a callousness score

The three subscales of the ICU formed the basis of our diagnostic approach. To address the particular requirements of the study, we additionally used two specific subscales of the PID-5 for defining a combined callousness score for each participant to increase the reliability of the measurement. We utilized the PID-5 subscales *Callousness* (14 items) and *Restricted Affectivity* (7 items). These facets show the closest resemblance to the ICU subscales. In our sample, the internal consistencies (Cronbach’s Alpha) of these two PID-5 facet scales were 0.90, and 0.85, respectively.

Although the three ICU- and the two PID-5 subscales were highly correlated (from r = 0.36 to 0.80), we did not simply add the standardized total scores of the subscales to get a combined callousness measure but calculated a principal component analysis. Results showed one main component with an eigenvalue greater than 1 (eigenvalue 3.31, explained variance 66,18%), indicating the highest positive associations with the ICU callous and the PID-5 callousness subscales (factor loadings of 0.89 and 0.86, respectively). The individual factor scores of this first component were then used as an individual callousness estimate to select the groups with the highest and lowest callousness scores.

### Stimulus material

To trigger orienting reactions, a series of 3 groups of 20 pictures each were presented on a personal computer, with 4 additional pictures for demonstrating the task. All pictures were obtained from the International Affective Picture System (IAPS)^50^. One group of 20 pictures depicted distress cues (i.e., pitiful scenes and persons such as crying children), one group depicted threatening images (e.g., threatening animals or facial expressions, guns, etc.), and the third group depicted images of neutral objects (e.g., a chair, a book). All pictures were selected based on normative valence and arousal ratings for the pictures available in the IAPS.

### Recording and quantification of heart rate responses

A simple and reliably measurable behavior of an individual is his/her response to novel stimuli, i.e., the so-called orienting response (OR). Several physiological components of the OR were well-characterized in a great variety of studies, which also indicated differences in certain characteristics associated with stimulus types and habituation effects^51^. With the presentation of affective pictures, heart rate changes have been proven as most suitable parameters to measure individual differences in the OR—they reliably indicate valence differences and show hardly any habituation effects in the first few seconds of viewing non-repeated, i.e., novel pictures^52^.

An electrocardiogram (ECG) was recorded using two adhesive electrodes (mounted on the left and right of the frontal chest wall just below the right clavicle and at the lower part of the left rib cage) and a portable ECG equipment. Continuous ECG monitoring was carried out with the eMotion Faros 180TM (Bittium Biosignals Ltd., Kuopio, Finland; single-channel ECG, Einthoven Lead II set-up, sampling rate 1 kHz; weight: 13 g; size: 48 × 29 × 12 mm). The raw data were converted from EDF to MATLAB^®^ data format for further analysis. To obtain heart rate time series with equidistant time steps, beat-to-beat values were re-sampled with 4 Hz using piecewise cubic spline interpolation after artifact correction^53^. Single artifacts were replaced by interpolation. Preceding the recording, the eMotion Faros and PC were synchronized to ensure synchronized stimulus presentation.

The ECG data in the time window beginning at picture onset and ending 6 s after picture onset were analyzed. Cardiac changes following the presentation of a picture were calculated relative to the heart rate immediately preceding the picture onset (mean of the 0.5 s frame). We attempted to increase the emotional valence of our pictorial stimulus material which should be included in the analyses. As all 3 × 20 pictures were subjectively evaluated for their emotional content by the participants, we eliminated five pictures each in one of the two emotional stimulus categories on the basis of these mean ratings. In the category of threatening pictures those five with the lowest ratings on the scale ‘makes me afraid’ were eliminated, and for the distress cues those five with the lowest ratings on the scale ‘makes me sad’ were removed. Additionally, five pictures from the neutral category were chosen and eliminated by random selection. Consequently, 3 × 15 pictures formed the basis of all data analyses. For each participant, the relative changes of cardiac variables were averaged across all trials of the two blocks for distress, threatening, and neutral cues, respectively. Also using IAPS pictures, Bradley and colleagues^52^ have demonstrated that specific stimulus-related heart rate deceleration takes place in the first three seconds after the onset of unpleasant (non-repeated) pictures. Consequently, we analyzed the time course of the transient cardiac responses for the first three seconds after picture onset.

### Experimental design

Before testing, written consent was obtained from each participant. After mounting the ECG electrodes and a resting period of 125 s, participants were informed that a series of pictures would be presented on the monitor in front of them. They were given the cover story that the experiment aimed to develop a psychological aptitude test to select applicants for a job as a photojournalist or a photographer who will be able to produce creatively and photographically outstanding work for newspaper reports or even for producing a catalog to advertise everyday objects. For this purpose, participants should rate the quality of each photo. As a very important additional note participants were told to form their opinion irrespective of the content of a picture, and just rate the photographic quality. Each picture would be presented for 6 s and, after that, a rating scale would be presented for 4 s to evaluate the quality of each photo with numbers ranging from 1 to 6 for indicating low to high quality. After three practice trials, the first part of the experiment started by presenting 30 pictures of the three valence categories in a randomized order. After a break of 30 s, the remaining 30 pictures were presented.

As a second task, all 60 pictures were presented again in the same randomized order as in the first block, but with the instruction to rate each picture on three different rating scales ranging from 1 to 6. Each picture was presented in the upper section of the screen, and in the lower part the rating scale was presented with the instruction to rate the picture according to three different criteria: “The picture makes me sad”, “The picture makes me furious”, and “The picture makes me afraid”. There was no time limit for these ratings.

### Statistical analyses

For evaluating the main research questions, a first three-way repeated measures ANOVA was done with the ECG changes as the dependent variable (see Results section: ‘ECG responses to stimuli’). The factorial design consisted of the three factors Group (2: individuals with low or high callousness scores) × Stimulus Type (3: threatening, distress, neutral) × Time (3: seconds after picture onset) to demonstrate the heart rate (HR) orienting response. In a second three-way repeated ANOVA the subjective ratings of the emotional impact of each picture were analyzed (see Results section: ‘Subjective evaluation of stimuli’). The factorial ANOVA design, however, was reduced to only two Stimulus Types, i.e., we did not include ratings of neutral pictures which showed variances close to zero and, therefore, would violate the assumptions of a mixed ANOVA. All statistical analyses were performed using SPSS 27.

## Results

For the two groups with low or high callousness scores, descriptive statistics are given in Table 1. They show about the same means of age (*t*(58) = 0.43, *p* = 0.67) but significant differences in the five subscales indicating a successful subdivision by the factor scores (one-way multivariate analysis of variance; *F*(5,54) = 81.06, *p* < 0.001, η_p_^2^ = 0.882). Moreover, mean heart rate and heart rate variability at rest were compared between groups. These analyses showed about the same heart rate (*M*_low_ = 75.11 bpm, *SD* = 9.96 vs. *M*_high_ = 75.99 bpm, *SD* = 10.27) for both study groups (*t*(58) = 0.33, *p* = 0.74). Heart rate variability also showed a non-significant difference between groups (*M*_low_ = 46.07, *SD* = 33.32 vs. *M*_high_ = 42.04, *SD* = 23.15; *t*(58) = 0.53, *p* = 0.59).

**Table 1:** Means and standard deviations for the total sample and the two groups separated by factor scores obtained by factoring three ICU- and two PID-5 subscales for callous-unemotional traits.

Variable	Total sample	Callousness scores		p
	n = 122	low (n = 30)	high (n = 30)	
	Mean (SD)	Mean (SD)	Mean (SD)	
Age	26.34 (4.94)	26.27 (4.54)	25.77 (4.45)	0.668
ICU: callous	7.85 (4.26)	4.03 (1.90)	13.07 (4.01)	< 0.001
ICU: uncaring	7.37 (3.53)	4.47 (2.20)	10.70 (3.74)	< 0.001
ICU: unemotional	7.89 (3.19)	4.37 (1.87)	10.88 (2.30)	< 0.001
PID-5: callousness	8.01 (6.86)	2.20 (1.73)	15.93 (7.47)	< 0.001
PID-5: restr. affectivity	8.21 (4.65)	3.40 (2.27)	13.67 (3.10)	< 0.001
Factor scores		− 1.15 (0.21)	1.34 (0.74)	

### ECG responses to stimuli

First, a significant main effect of Stimulus Type (*F*(2,57) = 7.05, *p* = 0.002, η_p_^2^ = 0.20) was observed indicating that threatening pictures resulted in a stronger HR deceleration compared to neutral ones (*M* = − 1.14, *SD* = 0.24 vs. *M* = − 0.54, *SD* = 0.23; *t*(59) = 3.67, *p* < 0.001). Moreover, distress cues also resulted in a more pronounced HR deceleration than neutral pictures (*M* = − 0.96, *SD* = 0.23 vs. *M* = − 0.54, *SD* = 0.23; *t*(59) = 2.21, *p* = 0.016). Second, a significant main effect of Time (*F*(2,57) = 15.12, *p* < 0.001, η_p_^2^ = 0.35) indicated increasing HR decelerations over the first three seconds (*M*_1_ = − 0.28, *SD* = 0.14; *M*_2_ = − 0.80, *SD* = 0.23; *M*_3_ = − 1.56, *SD* = 0.30). This main effect indicated the typical heart rate orienting response as a rapid, brief HR deceleration in response to the presented novel stimuli. No main effect was observed for Group (*F*(1,58) = 1.63, *p* = 0.207, η_p_^2^ = 0.03; *M*_low_ = − 1.15, *SD* = 0.30 vs. *M*_high_ = − 0.61, *SD* = 0.30). The most relevant result, however, relates to the significant interaction between Group by Stimulus Type (*F*(2,57) = 3.73, *p* = 0.030, η_p_^2^ = 0.12). Means and standard deviations of this two-way interaction are given in Table 2. The remaining two-way interactions Group by Time (*F*(2,57) = 0.265, *p* = 0.768) and Stimulus Type by Time (*F*(4,55) = 2.35, *p* = 0.065) as well as the three-way interaction Group by Stimulus Type by Time (*F*(4,55) = 1.04, *p* = 0.398) showed non-significant results.

**Table 2:** Means and standard deviations of ECG orienting responses (heart rate decelerations) to pictorial stimuli of the two groups with low and high callousness scores.

Stimulus type	Callousness scores		p	Cohen’s d
	Low (n = 30)	High (n = 30)		
	Mean (SD)	Mean (SD)		
Threatening	− 1.48 (0.34)	− 0.80 (0.34)	0.157	0.37
Distress cues	− 1.44 (0.32)	− 0.49 (0.32)	0.039	0.55
Neutral	− 0.53 (0.32)	− 0.55 (0.32)	0.959	0.01

Post-hoc analyses revealed that the groups differed in their ECG reactions to distress cues only. Participants with low callousness scores showed considerably greater HR decelerations than those with higher scores. A similar but statistically non-significant trend was observed for threatening pictures. Within-group effects demonstrated, on the one hand, that HR deceleration both to threatening and distress cues did not significantly differ from HR responses to neutral pictures within the group of high callousness scorers (simple effect within group: *F*(2,57) = 1.37, *p* = 0.261, η_p_^2^ = 0.05). On the other hand, participants with low callousness scores showed significantly larger reactions, i.e., greater HR decelerations both to threatening and distress cues compared to responses to neutral pictures (simple effect within group: *F*(2,57) = 9.41, *p* < 0.001, η_p_^2^ = 0.25; *t*(29) = 4.22, *p* < 0.001, Cohen’s *d* = 0.47 and *t*(29) = 3.61, *p* = 0.001, Cohen’s *d* = 0.49, respectively). Reactions to threatening and distress cues were nearly identical in this group.

### Subjective evaluation of stimuli

Means and standard deviations are given in Table 3 for participants with low callousness scores and in Table 4 for participants with high callousness scores, separately for each combination of 3 Stimulus Types by 3 Rating Categories. Although excluded from the factorial ANOVA for their extremely low variance, mean ratings of neutral pictures are also shown in Tables 3 and 4 for illustrative purposes.

**Table 3:** Mean ratings (standard deviations) of the emotional impact of stimuli on subjects with ‘*Low Callousness Scores*’ (N = 30) separated by Stimulus type and Rating category. Significant values are in bold.

Stimulus type	Rating category		
	Sad	Furious	Afraid
	Mean (SD)	Mean (SD)	Mean (SD)
Threatening	1.38 (0.10)	1.81 (0.13)	**3.16** (0.20)^a^
Distress cue	**4.30** (0.22)^b^	2.49 (0.17)	1.98 (0.14)
Neutral	1.05 (0.03)	1.02 (0.02)	1.04 (0.02)

**Table 4:** Mean ratings (standard deviations) of the emotional impact of stimuli on subjects with ‘*High Callousness Scores*’ (N = 30) separated by Stimulus type and Rating category.

Stimulus type	Rating category		
	Sad	Furious	Afraid
	Mean (SD)	Mean (SD)	Mean (SD)
Threatening	1.36 (0.10)	1.74 (0.13)	**2.49** (0.20)^a^
Distress cue	**3.10** (0.22)^b^	2.11 (0.17)	1.63 (0.14)
Neutral	1.03 (0.03)	1.02 (0.01)	1.01 (0.02)

Same superscript letters denote differences between scores in Table 3 and Table 4 identified by significant effects in respective simple ANOVAs. Higher means indicate higher emotional impact of stimuli. Significant values are in bold.

All three main effects proved significant, but only the main effect of Group (*F*(1,58) = 5.90, *p* = 0.018, η_p_^2^ = 0.09) is of some interest as individuals with low callousness scores gave higher ratings to both types of emotional stimuli across all rating categories (*M*_low_ = 2.51, *SD* = 0.13 vs. *M*_high_ = 2.05, *SD* = 0.13). All two-way interactions also showed significant effects, but again only the interaction between Stimulus Type and Rating Category (*F*(2,57) = 116.71, *p* < 0.001, η_p_^2^ = 0.80) is of some relevance, as it depicts the fact that threatening pictures received the highest “afraid” ratings, and distress cues the highest “sad” ratings (see Tables 3 and 4 for this effect). The really important issue of individual differences in the subjective ratings is reflected in the significant three-way interaction between Group by Stimulus Type by Rating Category (*F*(2,58) = 9.02, *p* < 0.001, η_p_^2^ = 0.14). A first important comparison concerns the ratings of threatening pictures as can be seen in comparing Tables 3 and 4. Participants with higher callousness scores indicated to be less afraid of the threatening stimuli than individuals with lower callousness scores (simple effect within threatening stimuli: *F*(1.42,58) = 3.68, *p* = 0.044, η_p_^2^ = 0.06; Greenhouse–Geisser corrected; see means in bold type and with superscript a in Tables 3 and 4). No substantial differences between groups are present in the rating categories “sad” and “furious” as far as threatening stimuli are concerned. Moreover, a second and also highly relevant comparison concerns the ratings of distress cues; as is also illustrated in Tables 3 and 4 participants with lower callousness scores showed higher “sad” ratings than individuals with higher callousness scores (simple effect within distress cues: *F*(1.72,58) = 8.03, *p* = 0.001, η_p_^2^ = 0.12; Greenhouse–Geisser corrected; see means in bold type and with superscript b in Tables 3 and 4). The furious and afraid ratings displayed no significant differences between groups in the evaluations of distress cues.

## Discussion

Two concepts have been considered as central foundations of the various and rather heterogeneous psychopathic traits. One is a strongly reduced emotionality and emotional empathy, and the other is a marked callousness, i.e., the prominent anti-social feature of psychopathy manifesting itself in a significant insensitivity to other's welfare and well-being^2,18^. The present study, therefore, was especially devoted to the investigation of the callous-unemotional trait, as a lack of responsiveness to distress cues may be seen as one of the core features of psychopathic individuals^36^. Furthermore, we tried to quantify this lack of responsiveness using heart rate responses as a sensible and valid parameter for measuring emotional reactivity to pictorial distress cues. As expected, the results clearly showed differences between the two study groups separated by questionnaires measuring callousness and restricted affectivity. These differences were seen in both the intensity of heart rate decelerations reflecting the immediate response to distress cues and in the individual ratings of the emotional impact of each picture. In line with expectations, participants with higher scores in callous-unemotional traits showed lower reactions to distress cues in HR responses and individual ratings than those with lower callousness scores. Moreover, heart rate responses were greater to distress cues than to neutral ones in the group with low callousness scores only. By contrast, heart rate decelerations were about the same size for distress cues and neutral stimuli in the group with higher callousness traits. This nearly identical reaction to distress and neutral pictures in the group with high callousness scores is especially remarkable as we tested and compared two non-forensic community samples with probably much smaller differences in the relevant personality characteristics compared to forensic versus non-forensic groups. In a largely comparable study, Blair and coworkers^36^ also observed a very small but nevertheless statistically significant difference in SCR responses to distress and neutral stimuli. As psychopathic and non-psychopathic delinquents were tested in that study, one might have expected even lower reactions to distress cues as in the present study. This might indicate higher sensitivity of cardiac as compared to electrodermal measures in this context.

In the present study, subjective ratings of pictorial stimuli’s emotional impact further supported the observations based on the ECG data. Mean ratings in the “sad” category which had turned out as most relevant for the evaluation of distress cues clearly differed between groups: participants with lower CU scores were significantly more affected than those with higher CU traits (see Tables 3 and 4; superscript b). In contrast to the ECG data, however, mean ratings of threatening stimuli also showed a significantly lower impact in the “afraid” rating category on individuals with higher CU scores (see Tables 3 and 4; superscript a). This result was expected on the basis of several studies on the psychopathic personality which reported deficiencies in the subjective experience of fear. According to a differentiated concept of fearlessness in psychopathy^40^, differences in subjective experience were clearly demonstrated in respective rating differences, whereas lower ECG responsivity in participants with higher callousness traits was not statistically substantiated. Similar results, for instance, were also reported recently in a sample of youth with disruptive behavior disorders^54^. Also using negative IAPS pictures (comparable to the items in the threatening category in the present study) participants in the clinical sample rated the negative images as less unpleasant than the control group, with the CU traits as the relevant predictor of participants’ emotional experience.

Apart from fearlessness, a lack of responsiveness to distress cues, i.e., the callous component of CU traits seems to constitute characteristics that generate central features of the psychopathic personality: the virtual absence of any conscience which in turn underlies the total lack of remorse or guilt, the criminal versatility, the failure to accept responsibility for own actions, and others. Consequently, the widely used PCL-R diagnostic, for instance, should be supplemented by testing CU traits. Moreover, except for the PCL-R, most published studies on psychopathic traits are based on questionnaire data, i.e., on self-reports about various aspects of participants’ own feelings and behavior. However, the physiological response of an individual to, for instance, the face of a suffering and crying child is possibly more to the core compared to any self-report data. In the callousness subscale of the ICU, for instance, one question is: ‘I am concerned about the feelings of others.’ Firstly, it must be assumed that the participant answers truthfully and, secondly, it must be assumed that he/she is really aware of his/her being affected by the feelings of others. Moreover, and especially in forensic settings, questionnaires may be of somewhat restricted validity as a particularly high amount of faking might be expected^55^. Therefore, some sort of simple but nevertheless valid measurements of a relevant physiological parameter would be most desirable. The experimental paradigm of the present study might be a possible template. However, Cohen’s d for the difference of means^56^ between the two groups indicated only a medium effect size of 0.55 (see Table 2) for the distress cues as stimulus type. Certainly, this is far from an effect size necessary for an individual diagnosis, but one could conceive various ways to improve the power of the paradigm. Such a neurophysiological supplement in diagnosing psychopathy may attenuate, for instance, most of the critical comments on PCL-R testing already cited in the introduction of the present paper.

However, the suggested approach has several limitations. Most importantly, the positive benefit of the physiological testing paradigm has yet to be replicated in samples of psychopathic individuals in criminal settings. Especially, in a further investigation a comparison of a community sample with forensic inmates with low and with high psychopathic traits should be realized as a particularly informative approach. Moreover, the selection of the community sample should also parallel the forensic samples for education and occupational backgrounds. Furthermore, our fake instruction to advertise our study might also have created a bias in participants who opted to take part in the study. After all, if sample sizes will be large enough in future studies, latent profile analysis will enable the identification of new subpopulations of psychopathic personalities on a statistical basis. Apart from the small sample size, we used a (non-criminal) community sample for the first evaluation of our physiological test paradigm. Although this may be seen as a further limitation, a large number of other studies on psychopathic traits were also investigating community samples like ours. Byrd and coworkers^43^, for instance, realized their validation study on the ICU on a large community sample of young adults living in the United States. Moreover, they collected a large number of validation criteria for psychopathic traits (self-reports of delinquency, psychopathy and psychopathology, official records of criminal charges, and others). Several of these variables were significantly related to the scores of the ICU subscales, even in their population-based community sample. Remarkably, the mean age and ICU scores of Byrd et al.’s sample were nearly identical to the sample characteristics of our study (see Table 1, first column). Thus, although our sample represents the lower end of the psychopathy continuum, it is comparable to the multitude of published studies on psychopathic traits which also focused on community samples. Importantly in this context, there is increasing evidence that psychopathic traits such as impulsivity, egocentricity, or emotion regulation display many of the same correlates in community samples as in prison samples^5,57,58^.

Although the measurement of ECG orienting responses was demonstrated as sensible enough to discriminate within a rather homogeneous group of individuals in the present study, the discrimination capability along the whole range of a trait must also be substantiated. Moreover, the actual validity of the ECG measures concerning the various facets of psychopathic personalities must yet be demonstrated. In the present study, we confined our interest to callousness, i.e., to one of the main components of CU traits. However, there is a variety of traits associated with psychopathic personalities. Consequently, a complex constellation of many facets may ultimately determine pathological behavior in criminal samples. Even in non-incarcerated community samples, the combination of psychopathic traits determined whether someone engages in criminal behavior or not^6^. That study aimed to identify distinct subgroups of adults based on their scores on three psychopathy dimensions by latent profile analysis. Additionally, several variables (e.g., various types of aggression and offence, substance abuse, internalizing problems like anxiety and depression) were obtained and their association with the latent profiles was analyzed. In addition to specific subgroups a psychopathic personality group was defined comprising persons scoring high in all subdimensions. Highly remarkable, results demonstrated that the combination of all psychopathy dimensions in this group showed the highest scores in all aggression and offence categories. However, there were a few exceptions (such as vandalism in men, which was specifically related to the impulsive-irresponsible dimension) indicating that assessing several different features may provide relevant clinical information that could be crucial for intervention efforts.

Therefore, future studies should also include self-report instruments such as the Triarchic Psychopathy Measure^59^ or the Psychopathic Personality Inventory-Revised^60^, but also the PCL-R itself to cover a broad range of validation criteria. Furthermore, in a comprehensive diagnosis of psychopathic traits ratings of various behavioral and emotional problems should also be included. Possibly, a multidimensional approach considering the various facets of the psychopathy construct may prove more efficient in the prediction of antisocial behaviors in prospective studies than an approach focusing solely on CU traits^61^. Finally, the retest-reliability of the testing procedure must also be thoroughly evaluated.

Nevertheless, a complementing assessment of psychopathy seems quite necessary, especially concerning a central concept of psychopathy, i.e., a lack of conscience, which manifests in an absence of remorse or guilt^62^. This facet will often have potentially severe consequences concerning judicial decisions^63^. Diagnostic efforts concerning psychopathic offenders’ conscience are further complicated by the fact that explicit tests of their moral understanding have very well revealed normal knowledge of wrongfulness and even comparatively normal responses in moral reasoning and moral foundation tests^64,65^. However, moral understanding and moral judgment are based on cognitive processes and, therefore, may be manipulated by psychopathic personalities’ intact theory of mind^55^. Yet, their emotional reactions to the suffering of their victims are different, i.e., they lack some sort of empathy for people in distress as a prerequisite for remorse and guilt. Consequently, a standardized procedure assessing physiological (and non-forgeable) reactions to cues indicating distress of others may be seen as most appropriate to capture one of the psychopathic individuals’ main deficiencies, i.e., callousness and lack of empathy.

The present study was confined to only one facet of the psychopathic personality. However, the used paradigm may well apply to other aspects of psychopathic and criminal personalities. As was recently suggested, research on the root ingredients of criminality should also include certain temperament variables like negative affectivity and self-control as essential determinants of antisocial behavior^66,67^. Recent empirical evidence obtained in a large sample of male prisoners supported the significance of such a temperament-and-character-based approach to the study of criminal and antisocial behavior^68^.

## Data Availability

The datasets generated during and/or analyzed during the current study are available from the corresponding author on reasonable request. The International Affective Picture System (IAPS) identification numbers of those pictures included in the data analysis are as follows. Distress cues: 2095 2278 2375 3181 3230 3300 3301 3350 9040 9041 9050 9250 9414 9432 9530. Threatening images: 0002 0003 0005 0007 0012 0015 1525 1726 1932 6230 6242 6250 6300 6370 6510. Neutral objects: 7002 7004 7006 7009 7025 7040 7050 7080 7090 7150 7175 7233 7235 7236 7705.
